# In Patients with Established RA, Positive Effects of a Randomised Three Month WBV Therapy Intervention on Functional Ability, Bone Mineral Density and Fatigue Are Sustained for up to Six Months

**DOI:** 10.1371/journal.pone.0153470

**Published:** 2016-04-13

**Authors:** Alessandra Prioreschi, Mohamed A. Makda, Mohammed Tikly, Joanne A. McVeigh

**Affiliations:** 1 Exercise Physiology Laboratory, School of Physiology, Faculty of Health Sciences, University of the Witwatersrand, Johannesburg, South Africa; 2 Department of Paediatrics, MRC/Wits Developmental Pathways for Health Research Unit, University of the Witwatersrand, Johannesburg, South Africa; 3 Division of Rheumatology, Department of Medicine, Chris Hani Baragwanath Academic Hospital, University of the Witwatersrand, Johannesburg, South Africa; University of Alabama at Birmingham, UNITED STATES

## Abstract

Functional ability is often impaired for people with rheumatoid arthritis (RA), rendering these patients highly sedentary. Additionally, patients with RA often take medication known to negatively affect bone mass. Thus improving functional ability and bone health in this group of patients is important. The aim of this study was to investigate the effects of whole body vibration (WBV) therapy in patients with stable, established RA. Thirty one females with RA were randomly assigned to a control group (CON, n = 15) who continued with their normal activities or a WBV group (n = 16) who underwent a three month WBV therapy intervention, consisting of 15 minutes of intermittent vibration, performed twice per week. Patients were assessed at baseline, three months, and three months post intervention for functional ability using the modified Health Assessment Questionnaire; for RA disease activity using the Clinical Disease Activity Index, for quality of life using self-report fatigue and pain scores; for physical activity profiles using accelerometry, and for BMD and body composition using DXA. Patients in both groups were matched for all variables at baseline. After the intervention period, functional ability was significantly improved in the WBV group (1.22(0.19) to 0.92(0.19), p = 0.02). Hip BMD was significantly reduced in the CON group (0.97(0.05) to 0.84(0.05) g.cm^-2^, p = 0.01), while no decreases were seen in the WBV group (1.01(0.05) to 0.94(0.05) g.cm^-2^, p = 0.50). Despite no change in RA disease activity in either group at either follow up, fatigue levels were improved in the WBV group (4.4(0.63) to 1.1(0.65), yet remained unchanged in the CON group at both follow ups (p = 0.01). Ten minute bouts of light to moderate physical activity were significantly reduced in the CON group after the intervention (2.8(0.61) to 1.8(0.64) bouts per day, p = 0.01), and were preserved in the WBV group (3.1(0.59) to 3.0(0.61) bouts per day, p = 0.70). Intermittent WBV shows promise for sustained improvements in functional ability, for attenuating loss of bone mass at the hip, as well as for decreasing fatigue in patients with established RA.

***Trial Registration*:** Pan African Clinical Trials Registry PACTR201405000823418

## Introduction

Rheumatoid arthritis (RA) is an autoimmune condition with a predilection for peripheral synovial joints. Inadequate or poor control of inflammation results in chronic pain, joint destruction, and decreased functional ability. Osteoporosis and osteopenia are common in patients with RA, and a variety of factors predispose RA patients to rapid loss of bone, including pro-inflammatory cytokines that augment osteoclast activity, certain medications taken to treat the disease such as oral corticosteroids, and decreased mobility. Levels of mobility and functional ability have been shown to be related to bone mineral density (BMD), as have age, stature, and sex, independently of RA disease [1], [2].

Moderate and vigorous habitual physical activities have known osteogenic effects [3]. Conversely, increased time spent in sedentary behaviours has been independently associated with poor bone mass in healthy populations [4]. Patients with RA spend the majority of their day being sedentary [5]. Furthermore, a large qualitative study conducted in female patients with RA found that patients reported pain and decreased functional ability as having the most widespread effect on their daily lives [6]. Inability to perform normal daily activities not only decreases quality of life, but also further perpetuates a sedentary lifestyle. Functional ability as assessed by the Health Assessment Questionnaire (HAQ), has been shown to be improved following certain exercise interventions conducted in patients with RA [7], yet there is still no consensus on the most feasible type of exercise intervention for patients with chronic pain and, often severe disability.

The beneficial effects of both aerobic and resistance exercise interventions to the health of patients with RA has been proven [8], yet exercise interventions in RA are not always feasible, specifically bone loading exercises which need to be dynamic in order to increase bone mass [9]. Whole body vibration (WBV) is safe, and requires minimal effort and movement [10]. The vibratory waves generated by a mechanical vibration platform produce energy via forced oscillation, which is then transferred to an individual via propagation through the feet, legs, trunk and the head [11]. WBV therapy has been shown to decrease fatigue and pain levels, and to improve muscle strength in patients with fibromyalgia [12], [13], and to decrease pain levels in patients with knee osteoarthritis [10], as well as to increase strength and balance, in various chronic conditions [14]. Furthermore, studies conducted in postmenopausal women [15], as well as in younger healthy populations [16], and athletes with low BMD [17] have shown WBV therapy to improve BMD, particularly at the hip and spine [18], [19].

WBV therapy has not, to the best of our knowledge, been used as a form of exercise in patients with RA. Furthermore, of the studies that have used WBV therapy in other populations, none have examined whether WBV therapy induced effects are sustained post intervention. The primary aim of this study was to determine the effects of a WBV programme on functional ability in patients with established RA. Secondly, the study aimed to determine any effects WBV therapy may have on BMD, RA disease activity, indices of health related quality of life (HRQoL), physical activity profiles, and body composition.

## Patients and Methods

### Study design

In this longitudinal study, consenting female patients were assigned (using an alternating method) to either a WBV group who underwent the WBV therapy, or a control (CON) group who continued with their normal daily activities. Following a baseline assessment (assessment 1), patients underwent the relevant intervention (WBV therapy or normal activity) for a three month period, following which all patients returned for a follow up assessment (assessment 2) in order to determine the effects of the WBV therapy intervention in comparison to the CON group. Thereafter, all patients continued with their normal activities for a further three months, following which a three month post intervention assessment was conducted (assessment 3) in order to determine whether any WBV therapy effects were sustained. Patients were enrolled from April to August 2013, the first follow up was completed for all participants by November 2013, and the final follow up for all participants was completed by the end of February 2014. The full protocol for this study has been published elsewhere [20]. The study protocol and CONSORT checklist can be found in the supporting information: S1 Text and S2 Text. The trial registration information for this study is PACTR201405000823418 (19/05/2014) and is available as S3 Text. This trial was only registered after data collection commenced as the protocol was first piloted for feasibility before registration.

### Patients

Thirty nine adult female patients who met the 1987 American College of Rheumatology classification criteria [21], had established disease of at least three years duration, and were on stable drug therapy (prednisone <10mg/day) for at least three months were included in the study. Patients were recruited and assessed at the Chris Hani Baragwanath Academic Hospital and gave written informed consent to be included in the study. Exclusion criteria were known HIV disease, use of bisphosphonates for the treatment of osteoporosis, any co-morbidities that could potentially impact on physical activity levels, use of assistive walking devices, previous hip or knee joint replacement surgery or arthroplasty, and pregnancy. Ethical approval was obtained from the Human Research Ethics Committee of the University of the Witwatersrand (M130113), and this trial conforms to the guidelines set out in the Declaration of Helsinki.

### Whole body vibration therapy intervention

Whole body vibration therapy consisted of two 15 minute session per week (total of 24 sessions over 12 weeks) of supervised therapy, which comprised of ten repetitions standing on the vibration plate for 60 seconds, followed by a 30 second rest period. This intermittent protocol was designed to increase osteogenic effects [22] and has previously been used successfully [17]. All WBV training was performed on the same vertical synchronous vibration plate (DKN XG 5.0, DKN Technology, California, USA). Different vibration platforms exist; vertical synchronous platforms move both legs up and down in unison, while oscillating plates move each leg up and down in a side-alternating manner. Vertical plates have most often been used to produce anabolic effects at the hip and spine as transmission of frequencies is site specific (vertical transmissions move in a vertical plane while side-alternating transmissions move in a more lateral plane); and accelerations produced are generally greatest at anatomical sites closest to the vibration platform [23]. Vibration was set at 3mm amplitude and a frequency of 30Hz in all instances, as lower amplitude, higher frequency vibrations have been shown to elicit greater responses in bone [24], and similar frequencies and amplitudes have been used in patients with fibromyalgia [14]. Patients were taught the correct posture (barefoot, holding on to the handle bars with knees slightly bent) while on the vibration plate in order to maximise the vibration effect while minimising any harmful effects, and to standardise procedure. All sessions were monitored by the primary investigator (AP) for compliance and accuracy. The CON group was instructed to continue with their normal daily activities for the three month period.

### Outcome assessments

#### Functional ability assessment

All patients were assessed at each time period for functional ability using the modified Health Assessment Questionnaire (mHAQ). The mHAQ is a well validated, self-administered, RA specific questionnaire that assesses functional ability in eight domains of daily activities where scores range from 0 (no disability) to 3 (severe disability) [25].

#### Disease activity and HRQoL measurements

All patients were assessed at each time point for rheumatoid disease activity using the Clinical Disease Activity Index (CDAI) by the same physician (MAM, who was blinded to the assignment of patients to either the WBV group or CON group). Patients were also asked to self assess their fatigue levels using a Lickert scale anchored at 0 (not tired at all) and 5 (the most tired I have ever felt), as well as their pain anchored at 0 (no pain) and 5 (unbearable pain).

#### Physical activity

Patients were fitted with an Actical (Respironics Inc., Murrysville, PA, USA) accelerometer worn on a Velcro belt on the right hip for a one week period at baseline, as well as at assessments 2 and 3 for the assessment of habitual physical activity. Patients then returned the accelerometer to the clinic one week later. Patients were instructed to wear the accelerometer all day, and to remove the device only while sleeping, bathing or showering. Actical data were recorded in one minute epochs and data were reduced by removing full days of non-wear time as observed by a full day of zero activity counts. Sleep time was removed by direct observation of the data [26], and only the remaining data were considered as wear time. Three days of wear time with a minimum of 10 hours of wear time per day was required for inclusion in the analysis [27].

#### Activity data

The Actical accelerometer records activity counts, which, based on the number of counts per epoch, are then classified by the inbuilt Actical software into thresholds of intensity; namely sedentary (0–100 counts), light activity (101–1485 counts), moderate activity (1486–5557 counts), and vigorous activity (≥5558 counts). Data were thus considered “active” data if a minute epoch had an activity count that was greater than 100 (i.e.: light to moderate/vigorous activity). Activity bouts were then calculated as the number of times per day that at least 10 consecutive minutes of “active data” were recorded [28]. The number of times that sedentary activity was “broken up” was calculated as the number of times per day that a sedentary minute epoch (<101 counts) was followed by at least one active minute epoch (>100 counts); and these data were thus considered as a break in sedentary time [29]. Data were reported as average activity counts per day; percentage of time spent in sedentary, light, moderate and vigorous activity thresholds; average number of activity bouts per day, as well as average number of sedentary breaks per day. Patients wore the same Actical at both visits in order to minimise inter-device variability.

#### BMD assessment

At each time point, patients underwent a Dual X-Ray Absorptiometry (DXA) scan to assess BMD and body composition. All patients were assessed for site specific areal BMD at the left hip, lumbar spine (L1-L4), and whole body. A single DXA operator was used throughout the study and was blinded as to the assignment of the patients to the WBV or CON group. A single machine was used (Hologic QDR 4500A, Hologic, Boston, USA), and was routinely calibrated throughout the study, using a phantom spine that was scanned daily to determine coefficients of variation of the machine. The coefficient of variation during the course of the study for spine BMD was 0.31%.

#### Body composition

At each time point, height (measured to the nearest mm using a stadiometer (Holtaine, UK)), and body mass (measured to the nearest 100g using a standard digital scale (Dismed, USA) were measured. From these measurements Body Mass Index (BMI) was calculated. Total lean and fat mass measurements were obtained from the body composition component of the whole body DXA scan. Skeletal muscle index (SMI) was calculated by adding appendicular lean mass (left and right- arm and leg- lean masses), and dividing by height squared [30].

### Statistical analysis

All analyses were conducted using Stata 12/IC 12.0 for Mac (StataCorp LP, College Station TX, USA) and p value of < .05 was considered statistically significant. A two sided sample size calculation for means with repeated measures (ß = 0.10) showed that at a 5% level (using an SD of 0.19) a sample of 8 patients in each group would be required in order to detect a minimum clinically important difference of 0.22 in mHAQ score [31] with a power of 90%. Students unpaired t-tests were used to compare continuous variables between the WBV group and CON group at baseline. Thereafter, to assess the effect of the intervention on the primary and secondary outcomes, individual linear mixed models were used for each dependent variable. Random intercepts were used to account for within person correlation of repeated measures and missing data as per an intention to treat analysis. To test a priori hypothesis, the estimated mean scores at each time point (three- and six months post baseline) were contrasted with baseline values for main effects, and group by time interactions as well as 95% confidence intervals (CI) were calculated. Model fit was assessed using residual plots and diagnostics. Percentage change from baseline was calculated and compared between groups using Students unpaired t-tests. All continuous variables are expressed as means and standard deviations (SD) or means and standard error of the means (SEM).

## Results

### Patient characteristics

**Fig 1:**
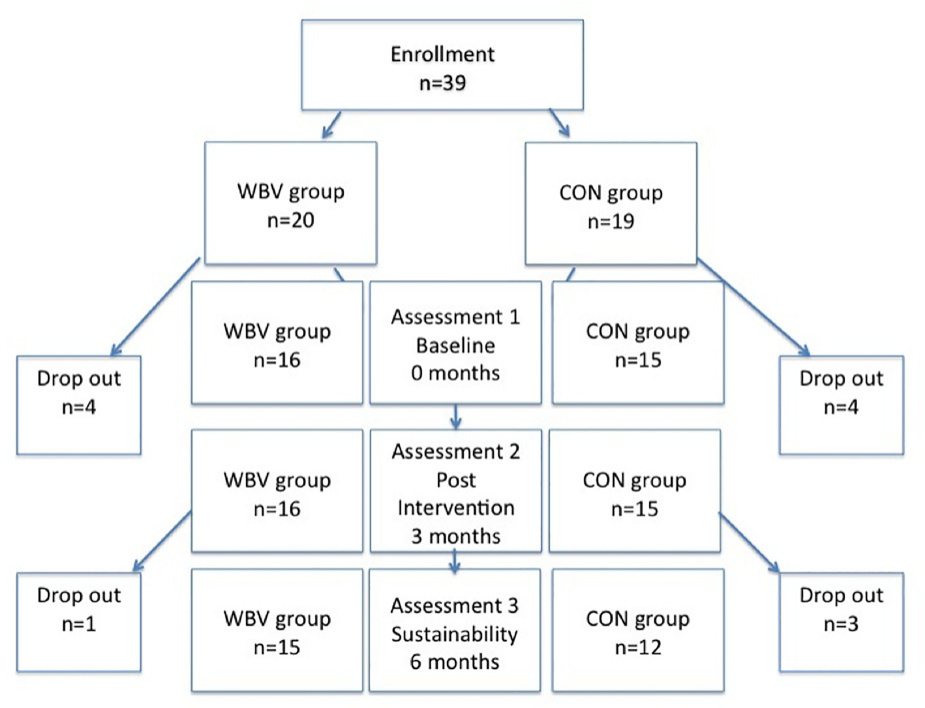
Number of study patients initially enrolled and followed up at each assessment time point.

Following enrollment, eight patients were excluded due to not being able to attend the required assessments. These patients were no different from completers in terms of age, disease duration, or functional ability (all p>0.05). Hence, 31 patients were allocated to the WBV group (n = 16) or the CON group (n = 15) and completed the three month intervention, however at assessment 3, one patient in the WBV group and three patients in the CON group were lost to follow up due to no longer being interested in participating, or not being able to attend the required assessments (Fig 1). Data can be found in the supporting information: S1 Data.

At baseline, there were no differences between the WBV group and the CON group for any of the variables measured as shown in Table 1. There were no differences between the WBV and CON groups for BMD at any of the sites measured at baseline (data shown in Table 2). Furthermore, there were no differences between the WBV and CON groups for average physical activity levels (p = 0.63, data shown in Table 2). Baseline (assessment 1), post intervention (assessment 2), and six month follow up (assessment 3) data for mHAQ, BMD, RA disease and physical activity variables are presented in Table 2.

**Table 1:** Patient characteristics at baseline in the WBV group and CON group. Data are mean (SD).

	WBV (n = 16)	CON (n = 15)	p value
Age (years)	51(10)	52(12)	0.81
Disease duration (years)	10(11)	12(8)	0.54
Height (m)	1.59(0.07)	1.55(0.08)	0.16
Body mass (kg)	85.24(21.96)	80.76(23.63)	0.59
CDAI	11(9)	8(6)	0.33
mHAQ (max. of 3)	1.22(0.67)	1.13(0.86)	0.74
Pain (max. of 5)	4(3)	4(3)	0.92
Fatigue (max. of 5)	4(3)	3(3)	0.35
Lean body mass (kg)	45.65(8.48)	42.11(7.96)	0.25
% body fat	42(7)	41(6)	0.55

CDAI–clinical disease activity index, mHAQ–modified health assessment questionnaire.

**Table 2:** Mixed model analysis between the WBV and CON groups at baseline, three- and six month assessments. Data are mean (SEM).

		WBV			CON			Mixed models analysis (p value)		
Outcomes	Variables	Assessment 1 (Baseline)	Assessment 2 (3 months)	Assessment 3 (6 months)	Assessment 1 (Baseline)	Assessment 2 (3 months)	Assessment 3 (6 months)	Group	Time	Group*time
**Functional Ability**	mHAQ (max. of 3)	1.22(0.19)	1.02(0.19)	0.92(0.19)*	1.13(0.20)	1.06(0.20)	1.17(0.20)	0.79	0.25	0.18
**HRQoL and Disease activity**	Fatigue (/5)	4.4(0.63)	1.1(0.65)*	3.7(0.67)	3.4(0.67)	3.7(0.75)	3.7(0.72)	0.40	0.04	0.01
	Pain (/5)	3.90(0.69)	3.07(0.71)	4.75(0.71)	4.00(0.71)	4.83(0.71)	4.75(0.78)	0.40	0.34	0.26
	CDAI	11.10(1.88)	9.59(1.88)	9.64(1.94)	8.20(1.94)	10.33(1.94)	8.80(1.94)	0.59	0.91	0.56
**Physical activity**	Average AC (/day)	7296(828)	7424(864)	7194(884)	6984(855)	6613(855)	6385(971)	0.53	0.85	0.88
	Sedentary (%/day)	68(2.6)	68(2.8)	70(2.8)	69(2.7)	70(2.8)	71(3.2)	0.67	0.57	0.96
	Light(%/day)	21(1.70)	21(1.80)	19(1.85)	20(1.75)	20(1.75)	20(2.08)	0.80	0.65	0.92
	Moderate (%/day)	11(1.27)	11(1.32)	10(1.35)	11(1.31)	10(1.31)	9(1.47)	0.59	0.50	0.91
	Vigorous (%/day)	0.02(0.01)	0.02(0.01)	0.01(0.01)	0.01(0.01)	0.01(0.01)	0.00(0.01)	0.31	0.75	0.91
	Breaks sedentary time (#/day)	61(3.3)	63(3.5)	63(3.6)	67(3.4)	60(3.4)*	61(4.0)	0.90	0.59	0.14
	Activity bouts (#/day)	3.1(0.59)	3.4(0.60)	3.0(0.61)	2.8(0.61)	2.1(0.61)*	1.8(0.64)*	0.26	0.14	0.16
**Bone**	Hip BMD (g.cm^2^)	1.01(0.05)	0.96(0.05)	0.94(0.05)	0.97(0.05)	0.94(0.05)	0.84(0.05)*	0.37	0.01	0.50
	Spine BMD (g.cm^2^)	0.92(0.04)	0.92(0.04)	0.92(0.04)	0.91(0.04)	0.91(0.04)	0.91(0.04)	0.87	0.84	0.74
	Whole Body BMD (g.cm^2^)	1.09(0.03)	1.10(0.03)^	1.10(0.03)*	1.07(0.03)	1.07(0.03)	1.05(0.03)*	0.47	0.15	<0.01
**Body Composition**	BMI (kg/m^2^)	34(2.1)	34(2.1)	33(2.1)*	34(2.2)	34(2.2)	34(2.2)	0.84	0.58	0.05
	Fat mass (kg)	37(3)	36(3)	35(3)*	31(3)	32(3)	35(3)*	0.43	0.44	<0.01
	Lean mass (kg)	46(2)	46(2)	45(2)	42(2)	42(2)	40(2)*	0.13	<0.01	<0.01
	SMI	7.8(0.39)	8.0(0.39)^	8.0(0.39)^	7.7(0.40)	7.9(0.40)*	7.4(0.41)*	0.64	0.02	<0.01

Breaks in sedentary time are adjusted for % total sedentary time at each time point.

mHAQ–modified health assessment questionnaire, HRQoL–health related quality of life, CDAI–clinical disease activity index, AC–activity counts, BMD–bone mineral density, BMI—body mass index, SMI–skeletal mass index.

* Main effects significantly different from baseline after posthoc analysis (p<0.05).

^ Main effects showed trend towards significant difference from baseline after posthoc analysis (p<0.07).

### Functional ability

**Fig 2:**
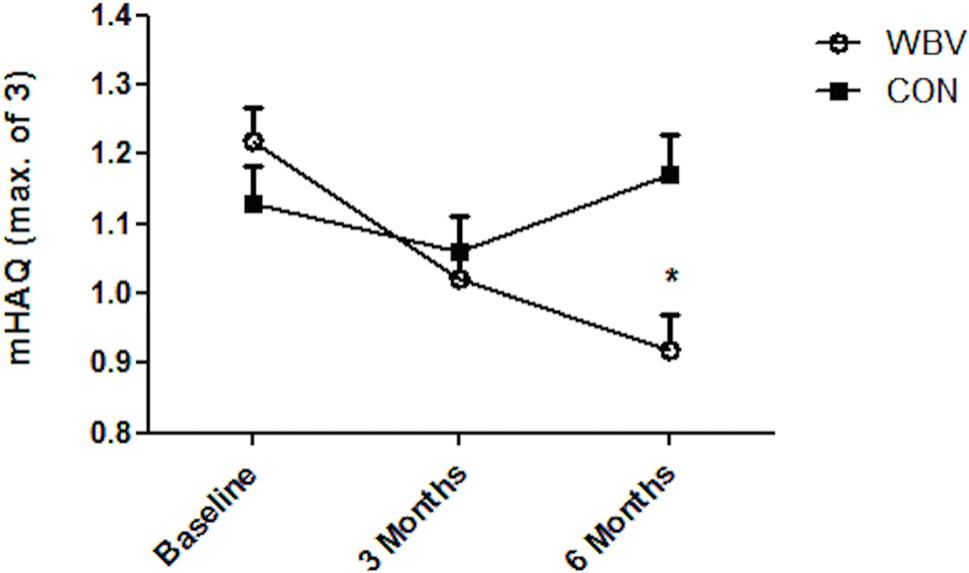
Change in functional ability. Assessed by the modified Health Assessment Questionnaire (mHAQ) from baseline to post intervention (three month assessment) and six month follow up between the whole body vibration group (WBV) and the control group (CON).

In the WBV group, mHAQ (where a lower score indicates an improved outcome) was improved (p = 0.02, CI -0.55 to -0.05) at assessment 3 as compared to baseline, while no changes were observed in the CON group at either assessment 2 or 3. Fig 2 shows the mean change in mHAQ between the two groups from baseline at each assessment. Of patients who underwent WBV therapy, 63% showed an improvement in mHAQ, while 60% of patients in the CON group showed a worsened mHAQ score over the three month intervention period.

### HRQoL and disease activity

**Fig 3:**
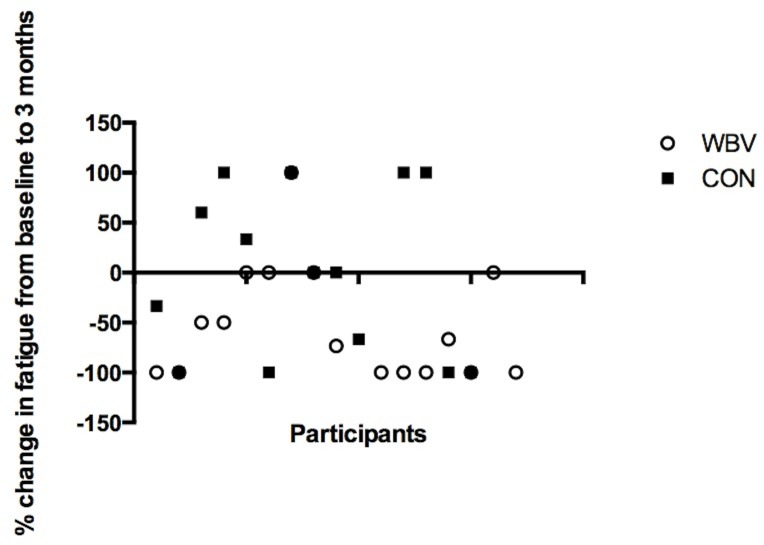
Percentage change in fatigue. From baseline to post intervention (three month assessment) for each participant in the whole body vibration group (WBV) and the control group (CON) where a negative change indicates less fatigue. Percentage change was greater in the WBV group (p = 0.06).

Table 2 shows the time effect (p = 0.04), as well as a group by time interaction (p = 0.01) for fatigue levels, where fatigue was decreased (p<0.01, CI -4.82 to -1.67) in the WBV group at assessment 2 as compared to baseline, however this effect was not sustained at assessment 3. No changes were observed in the CON group for fatigue levels over the study period. Fig 3 shows the percentage change in fatigue scores for each individual at the post intervention assessment as compared to baseline. Percentage change showed a trend towards being significantly greater in the WBV group compared to the CON group (p = 0.06). There were no changes in CDAI scores over the intervention period for either of the groups. There were no changes in pain levels over the study period for either of the groups.

### Physical activity

No main effects, or group by time interactions were observed for physical activity levels (Table 2). No changes were evident in either group for average activity counts per day, or percentage of time spent in sedentary, light, moderate or vigorous activity over the study period. However, in the CON group, there were fewer breaks in sedentary time (after adjusting for percentage of time in sedentary activity at each time point) (p = 0.05) at assessment 2 as compared to baseline. In the CON group, there was also a decrease (p = 0.05, CI -1.79 to -0.16) in number of activity bouts per day at assessment 2 as compared to baseline; and this effect was sustained at assessment 3 compared to baseline assessment (p = 0.01). No changes were observed in the WBV group for number of breaks in sedentary time, or number of active bouts.

### Bone mineral density

**Fig 4:**
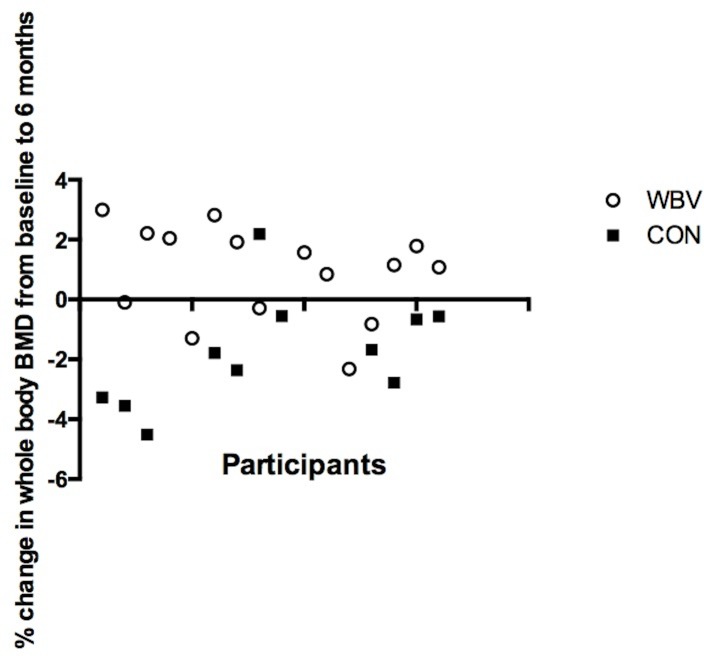
Percentage change in whole body bone mineral density. From baseline to six month follow up for each participant in the whole body vibration group (WBV) and the control group (CON) where a positive change indicates improved BMD. Percentage change was greater in the WBV group (p<0.001).

Hip BMD was lower (p = 0.01, CI -0.23 to -0.03) in the CON group at assessment 3 compared to baseline. No changes were observed for the WBV group. There were no changes in spine BMD over the study period for either of the groups. A time and group interaction was evident for whole body BMD (p<0.01), whereby there was a trend towards a significant (p = 0.06) increase in whole body BMD for the WBV group at assessment 2, and BMD was further increased at assessment 3 compared to baseline (p = 0.05, CI -0.01 to 0.01) (Table 2). In the CON group, whole body BMD was decreased (p<0.01, CI -0.02 to -0.002) at assessment 3 compared to baseline. When comparing the two groups at assessment 3, the WBV group showed improvement in whole body BMD by an average of (0.90(0.53)%) compared to the CON group, who on average lost 0.66(0.52)%, p = 0.04). Fig 4 shows percentage change in whole body BMD at six months compared to baseline for each participant. The WBV group showed sustained improvements, while the CON group showed sustained losses in whole body BMD (p<0.001).

### Body composition

Table 2 shows the group by time interaction effects for BMI (p = 0.05) and fat mass (p<0.01), both were decreased in the WBV group at assessment 3 as compared to baseline (p = 0.02, CI -1.20 to -0.08 and p = 0.01, CI -3794.93 to -416.50 respectively). In the CON group, fat mass increased (p<0.01, CI 1637.79 to 5830.64) at assessment 3 compared to baseline. Lean mass and SMI showed time effects (p<0.01, and p = 0.02 respectively), as well as group by time interactions (both p<0.01). Lean mass was decreased (p<0.01, CI -3363.64 to -1167.29) in the CON group at assessment 3 as compared to baseline. SMI showed a trend towards significant increase (p = 0.06) in the WBV group at assessment 2 from baseline, which was sustained at assessment 3 from baseline (p = 0.08), while in the CON group SMI was decreased (p<0.01, CI -1508.21 to -205.49) at assessment 2 from baseline, and at assessment 3 from baseline (p = 0.02, CI -1790.77 to -488.05).

## Discussion

The three month WBV therapy intervention in patients with RA had positive effects on functional ability, HRQoL, physical activity profiles, BMD and body composition. Importantly, mHAQ scores started to improve in patients who underwent the three month WBV therapy intervention, and were significantly improved at the six month follow up. Also of importance is the maintenance of whole body BMD observed in the WBV group after cessation of WBV therapy, as well as the attenuation of loss of hip BMD in the WBV group in comparison to the losses of hip BMD observed in the control group. The findings presented in the current lend support to the possibility that WBV therapy may be useful for increasing functional ability, as well as having a protective effect of bone health in patients with RA.

Improvements in activities of daily living as assessed by the mHAQ, is one of the key outcome measures of RA treatment [32]. The majority of patients (63%) who underwent WBV therapy showed either no change, or improvements in mHAQ scores. The overall improvement in mHAQ scores following the passive exercise programme used in the present study is comparable with those observed following active exercise interventions in groups of people with RA, which utilised either strength training [33], [34], or aerobic exercise programmes [35]. A WBV therapy intervention could therefore be a feasible means to improve the ability of these patients to perform their normal daily activities, thereby greatly increasing their sense of well being. WBV therapy has been shown to improve anti-inflammatory status in an elderly population [36], which may impact patient’s ability to function normally. Improvements in functional ability may also provide some protective effect on bone health in patients with RA, by allowing patients to be more mobile and thus potentially less sedentary.

Fatigue and ‘feeling well’ are important and relevant outcomes from the patient perspective in RA, and two of the most relevant improvements to note following treatment interventions in RA [32], [37]. Despite RA disease activity remaining unchanged following the WBV therapy intervention, fatigue levels were significantly improved by 53% (however these effects were not maintained at the six month follow up). The positive effects of WBV therapy on fatigue are similar to those observed in a cohort of patients with fibromyalgia who underwent a 6 week mixed WBV and exercise intervention where decreases in fatigue levels, as well as pain levels were observed, compared to a group that underwent exercise alone, and a control group where no changes were observed [12]. Another study conducted in patients with osteoarthritis also used a mixed exercise and WBV programme and were able to elicit improvements in pain levels over 8 weeks [10]. Moreover, WBV therapy in the present study has shown similar effects on well being as have aerobic and resistance exercise interventions in RA in previous studies [7], [35], [38].

Previous research has shown that higher levels of habitual physical activity in patients with RA are correlated with improved fatigue levels, as well as functional ability [5]. Recent literature has focused on the beneficial effects of breaking up sedentary time on health, regardless of overall activity levels [39], as well as the beneficial effects of being more active (often requiring activity to be performed in 10 minute bouts) [40]. Although overall physical activity remained unchanged before and after the intervention for both groups, there were important changes observed in the patterns of physical activity that should be considered. The CON group decreased the number of times they broke up their sedentary time and also had fewer bouts of activity following the intervention signifying more time spent in prolonged period of uninterrupted sedentary time in this group, yet activity profiles were maintained in the WBV group throughout the intervention period. Limiting sedentary time has been shown to have beneficial effects on bone health [4]. It is possible that the WBV therapy intervention, by means of decreasing fatigue levels, allowed patients to modify their habitual physical activity profiles and to start engaging in less sedentary behaviours, thereby becoming more functional.

In an RA population, where bone health is often compromised and increasing the risk of fractures, maintenance of BMD is an important secondary goal of treatment. In the present study it was found that WBV therapy improved whole body BMD in most patients (non significant average 8% increase from baseline), and that this effect was carried forward for a further three months (significant average 9% increase from baseline). WBV therapy also preserved hip BMD levels, while the CON group experienced losses in BMD at both the hip (-13%) and whole body (-1%) over the six month period. The loss of hip BMD in the CON group was greater than the least significant change needed for clinical relevance of 0.02g.cm^2^ at the hip (0.04g.cm^2^ is needed at the spine [41]). As expected in a group of women with RA, the WBV group also experienced clinically relevant decreases in hip BMD, yet these were much smaller and non significant. Healthy participants of a similar age range have been shown to lose 3% BMD at the hip over one year [42], and the NHANES database values for healthy women between 40–50 years shows approximately 5% decrease over 10 years [43]. Hip BMD has been shown to be the best predictor of fracture risk at any site [44], and WBV therapy may thus be considered as an adjunct therapeutic option for counteracting the very rapid loss of BMD that occurs in women with RA.

We, and others, have previously shown that WBV therapy improves or maintains BMD to varying degrees, particularly at the hip and spine. Versheuren et al in 2004 [19] and Rubin et al in 2004 [18] showed that WBV training for a six month and a one year period respectively, was able to significantly increase hip BMD by up to 0.93% (and 1.37% in only compliant participants) in a group of postmenopausal women. Rubin and colleagues further showed improvements in spine BMD of 0.49% in the compliant subset of their cohort. Prioreschi et al in 2012 were also able to show increases in hip BMD of 1.65%, and attenuated losses of spine BMD in a group of road cyclists who participated in only 10 weeks of intermittent WBV therapy [17]. Some studies have found no effects of WBV therapy on bone [23], however confounding variables such as Vitamin D and calcium supplementation in the control group, young age of participants, use of oscillating platforms, and lack of an intermittent protocol could explain the lack of osteogenic effects observed in these studies. Site specific differences in BMD changes evident between studies may also be due to transmission of frequencies along differing axes due to the type of vibration plate used, and the amount of acceleration produced dependant on frequency and amplitude settings. WBV therapy was well tolerated by the present cohort, and could potentially be a feasible intervention for BMD in patients with RA that does not require the vigorous, high-impact movements that are usually required to increase BMD (yet are often not feasible in this population). Although the exact mechanisms whereby WBV increases BMD remain unclear and depend on the frequencies and amplitudes utilised, it is likely that there are multiple mechanisms at play. Whole body vibration has been shown to activate fluid flow in the caniliculi and lacunae of bone matrix in rats [45], in a manner proportional to loading frequency, creating a shear stress on the plasma membrane of osteocytes, bone lining cells, and osteoblasts, which may respond accordingly [9]. However, Uzer et al., (2013) showed that *in vitro* fluid shear did not regulate vibration induced proliferation, and that cytoskeletal actin remodeling may play a greater role [46]. WBV activates mechanotransduction in bone and can stimulate osteogenesis [45], and low magnitude, high frequency vibration has been shown *in vitro* to upregulate osteoblast differentiation, matrix synthesis and mineralization, while regulating osteoclastic activity [24]. Furthermore, muscle forces exert osteogenic stimulus on bone, and the generation of these forces through vibration stimulus is a likely contributor to the skeletal adaptations that occur [47].

Although the frequency used in this study was chosen to achieve osteogenic effects, WBV therapy in this study also had a positive impact on body composition, lending support to the use of frequencies of 30Hz for affecting body composition. The WBV group in this study lost a significant amount of body mass (specifically -5% body fat) during the intervention period, maintained their lean muscle mass, and showed a trend towards improving SMI (an indicator of sarcopenia), while both SMI and lean mass were decreased in the CON group by 5% and 4% respectively. Sarcopenia, in combination with osteoporosis, greatly increases the risk of obtaining a fracture [48]. Whole body vibration has previously been shown to decrease BMI, and body fat (as measured by DXA) in obese women [49], to improve muscle strength in women with fibromyalgia [15], and to slow fat acquisition in rodent models [50]. This may be related to the ability of WBV therapy to increase oxygen consumption, energy expenditure, and neuromuscular performance [51] through continuously effecting eccentric and concentric muscular contractions [49], however higher frequencies are usually needed to elicit these effects. It is possible that the relatively low levels of habitual activity in this cohort allowed for changes in energy expenditure and thus body composition, even with lower frequencies of vibration. Alternatively, the simple participation in an organised activity twice weekly may have result in greater energy expenditure than normal. In both of the aforementioned studies a decrease in fat mass following WBV was mirrored by an increase in BMD or bone mineral content, which was not observed in the relevant control group. Although lower body mass is a known risk factor for low bone mass [52], and it is therefore assumed that higher body mass is protective of BMD, recent studies have suggested that obesity may have an inverse relationship with BMD [53], and that fractures that occur in obese populations should be considered ‘fragility fractures’ [54]. Potentially, obesity is distinct from ‘high body mass’ in terms of the effect it has on bone health; and should still be avoided in patients with RA, as is recommended in the general population. These results suggest that WBV therapy may be useful in decreasing body mass, as well as increasing lean muscle mass in patients with RA.

This study has strengths and limitations. The results showed that many of the benefits of WBV therapy in patients with RA can be sustained for at least three months post therapy. Furthermore, attrition rates were low, suggesting that the WBV program was well tolerated within this population. Unfortunately no qualitative assessments of the intervention were made, and thus the possibility that group participation had an effect on various outcomes cannot be negated. Since the intervention ran over a year period with staggered recruitment, the effects of the colder temperatures in winter may have altered various pain and well-being outcomes; however patients were recruited in an alternating manner, which would limit the potential differences between groups. Vitamin D, calcium intake and menopausal status were unfortunately not controlled for, which may have confounded changes in bone density. Furthermore, the small sample size and limited duration of the intervention must be considered.

In conclusion, the findings of this study show that a WBV therapy intervention can illicit sustained improvements in functional ability in females with RA. Bone mass at the hip and whole body was preserved, as was lean muscle mass. Patients who participated in the WBV therapy intervention also had improved fatigue levels without any changes in RA disease activity being observed. Furthermore, patients in the WBV group maintained their habitual physical activity profiles throughout the assessment period. Based on these findings, it seems that WBV therapy offers a useful and affordable adjunct therapy with sustained effects for patients with established RA, which does not require vigorous, high-impact movements. Future studies including qualitative assessments need to be conducted over a longer duration in a larger cohort of both males and females.

## Supporting Information

S1 Data Spreadsheet of raw data. (XLSX)

S1 Text Study Protocol. (DOCX)

S2 Text CONSORT Checklist. (PDF)

S3 Text Trial registration. (PDF)

## References

[1] Laan RF, Buijs WC, Verbeek AL. Bone mineral density in patients with recent onset rheumatoid arthritis: influence of disease activity. Ann Rheum Dis. 1993;52:21–6. 8427509 10.1136/ard.52.1.21 PMC1004950

[2] Deodhar AA, Woolf AD. Bone mass measurement and bone metabolism in rheumatoid arthritis: a review. Br J Rheumatol. 1996;35:309–22. 8624634 10.1093/rheumatology/35.4.309

[3] Ozcivici E, Luu YK, Adler B, Qin Y-X, Rubin J, Judex S, et al Mechanical signals as anabolic agents in bone. Nat Rev Rheumatol. Nature Publishing Group; 2010;6:50–9. 10.1038/nrrheum.2009.239 20046206 PMC3743048

[4] Tremblay MS, Colley RC, Saunders TJ, Healy GN, Owen N. Physiological and health implications of a sedentary lifestyle. Appl Physiol Nutr Metab. 2010;35:725–40. 10.1139/H10-079 21164543

[5] Prioreschi A, Hodkinson B, Avidon I, Tikly M, McVeigh JA. The clinical utility of accelerometry in patients with rheumatoid arthritis. Rheumatology (Oxford). 2013 26;53:1–7. 10.1093/rheumatology/ket216 23804220

[6] Schneider M, Manabile E, Tikly M. Social aspects of living with rheumatoid arthritis: a qualitative descriptive study in Soweto, South Africa—a low resource context. Health Qual Life Outcomes. 2008;6:54 10.1186/1477-7525-6-54 18651986 PMC2499996

[7] Metsios GS, Stavropoulos-Kalinoglou A, Veldhuijzen van Zanten JJCS, Treharne GJ, Panoulas VF, Douglas KMJ, et al Rheumatoid arthritis, cardiovascular disease and physical exercise: a systematic review. Rheumatology (Oxford). 2008;47:239–48. 18045810 10.1093/rheumatology/kem260

[8] Cooney JK, Law R-J, Matschke V, Lemmey AB, Moore JP, Ahmad Y, et al Benefits of exercise in rheumatoid arthritis. J Aging Res. 2011; 10.4061/2011/681640 PMC3042669 21403833

[9] Turner CH, Robling AG. Exercises for improving bone strength. Br J Sports Med. 2005;39:188–9. 15793082 10.1136/bjsm.2004.016923 PMC1725178

[10] Park YG, Kwon BS, Park J-W, Cha DY, Nam KY, Sim KB, et al Therapeutic effect of whole body vibration on chronic knee osteoarthritis. Ann Rehabil Med. 2013;37:505–15. 10.5535/arm.2013.37.4.505 24020031 PMC3764345

[11] Wysocki A, Butler M, Shamliyan T, Kane RL. Review Annals of Internal Medicine Whole-Body Vibration Therapy for Osteoporosis: State of the Science. 2011;155. 10.7326/0003-4819-155-10-201111150-00006 22084334

[12] Alentorn-Geli E, Padilla J, Moras G, Lázaro Haro C, Fernández-Solà J. Six weeks of whole-body vibration exercise improves pain and fatigue in women with fibromyalgia. J Altern Complement Med. 2008;14:975–81. 10.1089/acm.2008.0050 18990045

[13] Sañudo B, Hoyo M De, Carrasco L, Mcveigh JG, Corral J, Cabeza R, et al The effect of a 6-week exercise programme and whole body vibration on strength and quality of life in women with fibromyalgia: a randomised study. Clin Exp Rheum. 2010;28:S40–45. 21122265

[14] Pang MY. Whole body vibration therapy in fracture prevention among adults with chronic disease. World J Orthop. 2010;1:20–5. 10.5312/wjo.v1.i1.20 22474623 PMC3302025

[15] Bemben DA, Palmer IJ, Bemben MG, Knehans AW. Effects of combined whole-body vibration and resistance training on muscular strength and bone metabolism in postmenopausal women. Bone. 2010;47:650–6. 10.1016/j.bone.2010.06.019 20601282

[16] Cardinale M, Wakeling J. Whole body vibration exercise: are vibrations good for you? Br J Sports Med. 2005;39:585–9. 16118292 10.1136/bjsm.2005.016857 PMC1725325

[17] Prioreschi A, Oosthuyse T, Avidon I, Mcveigh J. Whole Body Vibration Increases Hip Bone Mineral Density in Road Cyclists. Int J Sports Med. 2012;33:593–9. 10.1055/s-0032-1301886 22562741

[18] Rubin C, Recker R, Cullen D, Ryaby J, McCabe J, McLeod K. Prevention of postmenopausal bone loss by a low-magnitude, high-frequency mechanical stimuli: a clinical trial assessing compliance, efficacy, and safety. J Bone Miner Res. 2004;19:343–51. 15040821 10.1359/JBMR.0301251

[19] Verschueren SMP, Roelants M, Delecluse C, Swinnen S, Vanderschueren D, Boonen S. Effect of 6-month whole body vibration training on hip density, muscle strength, and postural control in postmenopausal women: a randomized controlled pilot study. J Bone Miner Res. 2004;19:352–9. 15040822 10.1359/JBMR.0301245

[20] Prioreschi A, Tikly M, McVeigh JA. A three month controlled intervention of intermittent whole body vibration designed to improve functional ability and attenuate bone loss in patients with rheumatoid arthritis. BMC Musculoskelet Disord. 2014 29;15:403 10.1186/1471-2474-15-403 25433517 PMC4265489

[21] Arnett FC, Edworthy SM, Bloch DA, McShane DJ, Fries JF, Cooper NS, et al The American Rheumatism Association 1987 revised criteria for the classification of rheumatoid arthritis. Arthritis Rheum. 1988;31:315–24. 3358796 10.1002/art.1780310302

[22] Turner CH. Three rules for bone adaptation to mechanical stimuli. Bone. 1998;23:399–407. 9823445 10.1016/s8756-3282(98)00118-5

[23] Beck BR. Vibration Therapy to Prevent Bone Loss and Falls: Mechanisms and Efficacy. Curr Osteoporos Rep. 2015;13:381–9. 10.1007/s11914-015-0294-8 26456496

[24] Zhou Y, Guan X, Liu T, Wang X, Yu M, Yang G, et al Whole body vibration improves osseointegration by up-regulating osteoblastic activity but down-regulating osteoblast-mediated osteoclastogenesis via ERK1/2 pathway. Bone. 2015;71:17–24. 10.1016/j.bone.2014.09.026 25304090

[25] Bruce B, Fries JF. The Health Assessment Questionnaire (HAQ). Clin Exp Rheumatol. 2005;23:14–8. 16273780

[26] Kinder J, Lee K, Thompson H, Hicks K, Topp K, Madsen KA. NIH Public Access. J Paediatr Nurs. 2013;27:127–33.

[27] Ellingson L, Colbert L, Cook D. Physical Activity Is Related to Pain Sensitivity in Healthy Women. Med Sci Sport Exerc. 2012:1401–6. 10.1249/MSS.0b013e318248f648 22217571

[28] Tudor-Locke C, Camhi SM, Troiano RP. A catalog of rules, variables, and definitions applied to accelerometer data in the National Health and Nutrition Examination Survey, 2003–2006. Prev Chronic Dis. 2012;9:110332. 10.5888/pcd9.110332 PMC3457743 22698174

[29] Tudor-locke C, Brashear MM, Johnson WD, Katzmarzyk PT. Accelerometer profiles of physical activity and inactivity in normal weight, overweight, and obese U.S. men and women. Int J Behav Nutr Phys Act. 2010;7:60 10.1186/1479-5868-7-60 20682057 PMC2924256

[30] Figueiredo CP, Domiciano DS, Lopes JB, Caparbo VF, Scazufca M, Bonfá E, et al Prevalence of sarcopenia and associated risk factors by two diagnostic criteria in community-dwelling older men: the São Paulo Ageing & Health Study (SPAH). Osteoporos Int. 2014;25:589–96. 10.1007/s00198-013-2455-x 23892584

[31] Redelmeier D, Lorig K. Assessing the clinical importance of symptomatic improvements. An illustration in rheumatology. Arch Intern Med. 1993;153:1337–42. 8507124

[32] Klareskog L, Catrina AI, Paget S. Rheumatoid arthritis. Lancet. 2009;373:659–72. 10.1016/S0140-6736(09)60008-8 19157532

[33] McMeeken J, Stillman B, Story I, Kent P, Smith J. The effects of knee extensor and flexor muscle training on the timed-up-and-go test in individuals with rheumatoid arthritis. Physiotherapy research international. 1999;4:55–67. 10368839 10.1002/pri.1999.4.1.55

[34] Hakkinen A, Sokka T, Kautianinen H, Kotaniemi A, Hannonen P. Sustained maintenance of exercise induced muscle strength gains and normal bone mineral density in patients with early rheumatoid arthritis: a 5 year follow up. Ann Rheum Dis. 2004;63:910–7. 15249317 10.1136/ard.2003.013003 PMC1755099

[35] Baillet A, Zeboulon N, Gossec L, Combescure C, Bodin L-A, Juvin R, et al Efficacy of cardiorespiratory aerobic exercise in rheumatoid arthritis: meta-analysis of randomized controlled trials. Arthritis Care Res. 2010;62:984–92. 10.1002/acr.20146 20589690

[36] Rodriguez-Migueleza P, Fernandez-Gonzalob R, Colladoc PS, Almarc M, Martinez-Florezc S, Pazc JA de, et al Whole-body vibration improves the anti-inflammatory status in elderly subjects through toll-like rec. Mech Aging Dev. 2015;150:12–9. 10.1016/j.mad.2015.08.002 26253933

[37] Sanderson T, Morris M, Calnan M, Richards P, Hewlett S. Patient perspective of measuring treatment efficacy: the rheumatoid arthritis patient priorities for pharmacologic interventions outcomes. Arthritis Care Res. 2010;62:647–56. 10.1002/acr.20151 PMC2886964 20461786

[38] Cairns AP, McVeigh JG. A systematic review of the effects of dynamic exercise in rheumatoid arthritis. Rheumatol Int. 2009;30:147–58. 10.1007/s00296-009-1090-5 19701638

[39] Healy GN, Matthews CE, Dunstan DW, Winkler EAH, Owen N. Sedentary time and cardio-metabolic biomarkers in US adults: NHANES 2003–06. Eur Heart J. 2011;32:590–7. 10.1093/eurheartj/ehq451 21224291 PMC3634159

[40] Pate RR, O’Neill JR, Lobelo F. The evolving definition of “sedentary”. Exerc Sport Sci Rev. 2008;36:173–8. 10.1097/JES.0b013e3181877d1a 18815485

[41] El Maghraoui A, Roux C. DXA scanning in clinical practice. QJM. 2008; 101:605–17. 10.1093/qjmed/hcn022 18334497

[42] Bainbridge KE. Natural History of Bone Loss over 6 Years among Premenopausal and Early Postmenopausal Women. Am J Epidemiol. 2002;156:410–7. 12196310 10.1093/aje/kwf049

[43] Looker A, Borrud LG, Highes JP. Lumbar Spine and Proximal Femur Bone Mineral Density, Bone Mineral Content, and Bone Area. United States, Vital Heal stat. 2012;11:1–141. 24261130

[44] Leslie WD, Lix LM, Tsang JF, Caetano PA. Single-Site vs Multisite Bone Density Measurement for Fracture Prediction. Arch Intern Med. 2007;167:1641–7. 17698687 10.1001/archinte.167.15.1641

[45] Totosy de Zepetnek JO, Giangregorio LM, Craven BC. Whole-body vibration as potential intervention for people with low bone mineral density and osteoporosis: A review. J Rehabil Res Dev. 2009;46:529 19882487 10.1682/jrrd.2008.09.0136

[46] Uzer G, Pongkitwitoon S, Ete Chan M, Judex S. Vibration induced osteogenic commitment of mesenchymal stem cells is enhanced by cytoskeletal remodeling but not fluid shear. J Biomech. 2013;46:2296–302. 10.1016/j.jbiomech.2013.06.008 23870506 PMC3777744

[47] Rittweger J. Vibration as an exercise modality: how it may work, and what its potential might be. Eur J Appl Physiol. 2010;108:877–904. 10.1007/s00421-009-1303-3 20012646

[48] Burini RC. Sarcopenia in rheumatoid cachexia: definition, mechanisms, clinical consequences and potential therapies. Bras J Rheumatol. 2009;49:288–301

[49] Milanese C, Piscitelli F, Zenti MG, Moghetti P, Sandri M, Zancanaro C. Ten-week whole-body vibration training improves body composition and muscle strength in obese women. Int J Med Sci. 2013;10:307–11. 10.7150/ijms.5161 23423629 PMC3575626

[50] Maddalozzo G, Iwaniec U, Turner R, Rosen C, Widrick J. NIH Public Access. Int J Obes. 2009;32:1348–54. 10.1038/ijo.2008.111 PMC2586051 18663370

[51] Tapp LR, Signorile JF. Efficacy of WBV as a modality for inducing changes in body composition, aerobic fitness, and muscular strength: a pilot study. Clin Interv Aging. 2014;9:63–72. 10.2147/CIA.S30048 24399871 PMC3875193

[52] Laan M. Bone mineral density in patients with recent onset rheumatoid arthritis: influence of disease activity. Ann Rheum Dis. 1993;52:21–6. 8427509 10.1136/ard.52.1.21 PMC1004950

[53] Bredella M, Torriani M, Ghomi R, Thomas B, Brick D, Gerweck AV, et al Determinants of bone mineral densty in obese premenopausal women. Bone. 2012;48:748–54. 10.1016/j.bone.2010.12.011 PMC3073669 21195217

[54] Premaor MO, Ensrud K, Lui L, Parker RA, Cauley J, Hillier TA, et al Risk factors for nonvertebral fracture in obese older women. J Clin Endocrinol Metab. 2011;96:2414–21. 10.1210/jc.2011-0076 21677038 PMC3146794

