# Supplementary material for: In Patients with Established RA, Positive Effects of a Randomised Three Month WBV Therapy Intervention on Functional Ability, Bone Mineral Density and Fatigue Are Sustained for up to Six Months
Source: PLoS One. 2016 Apr 13;11(4):e0153470. doi: 10.1371/journal.pone.0153470 (PMC4830593; doi:10.1371/journal.pone.0153470)
Supplement: S1 Text — (DOCX) [file pone.0153470.s002.docx]

**A three month controlled intervention of intermittent whole body vibration designed to improve functional ability and attenuate bone loss in patients with rheumatoid arthritis**

Alessandra Prioreschi^1^, Mohammed Tikly^2^, Joanne A McVeigh^1^

1. Exercise Physiology Laboratory, School of Physiology, Faculty of Health Sciences, University of the Witwatersrand, Johannesburg, South Africa
2. Division of Rheumatology, Department of Medicine, Chris Hani Baragwanath Academic Hospital, University of the Witwatersrand, Johannesburg, South Africa

Abstract:

**Background:** Rheumatoid arthritis (RA) is a chronic autoimmune condition that results in pain and disability. Patients with RA have a decreased functional ability and are forced into a sedentary lifestyle and. As such, these patients often become predisposed to poor bone health. Patients with RA may also experience a decreased health related quality of life (HRQoL) due to their disease. Whole body vibration (WBV) is a form of exercise that stimulates bone loading through forced oscillation. WBV has also been shown to decrease pain and fatigue in other rheumatic diseases, as well as to increase muscle strength. This paper reports on the development of a semi randomised controlled clinical trial to assess the impact of a WBV intervention aiming to iprove functional ability, attenuate bone loss, and improve habitual physical activity levels in patients with RA. **Methods and Design:** This study is a semi randomised, controlled trial consisting of a cohort of patients with established RA assigned to either a WBV group or a CON (control) group. Patients in the WBV group will undergo three months of twice weekly intermittent WBV sessions, while the CON group will receive standard care and continue with normal daily activities. All patients will be assessed at baseline, following the three month intervention, and six months post intervention. Main outcomes will be an improvement in functional ability as assessed by the HAQ. Secondary outcomes are attenuation of loss of bone mineral density (BMD) at the hip and changes in RA disease activity, HRQoL, habitual physical activity levels and body composition.

**Discussion:** This study will provide important information regarding the effects of WBV on functional ability and BMD in patients with RA, as well as novel data regarding the potential changes in objective habitual physical activity patterns that may occur following the intervention. The sustainability of the intervention will also be assessed.

Introduction:

**Rheumatoid Arthritis (RA) is the most common autoimmune disease**. RA causes joint swelling, tenderness and destruction of the synovial joints. It is a chronic condition resulting in pain, severe disability and decreased functional ability (1). RA occurs in 1% of people worldwide, with the prevalence being two times higher in women than in men (2).

**People living with RA have decreased functional ability.** The health assessment questionnaire (HAQ), which is a disease specific measure of functional ability in RA, is one of the most increasingly and widely used outcome measures for RA (3). A large qualitative study conducted in female patients with RA found that patients report pain and decreased functional ability as having the most widespread effect on their daily lives (4). Inability to perform normal daily activities not only decreases quality of life, but also further perpetuates a sedentary lifestyle in these patients. Certain exercise interventions conducted on patients with RA have managed to significantly improve functional ability as assessed by the HAQ through increasing participation in regular physical activity (5).

**People living with RA are predisposed to the development of osteoporosis.** Patients with RA have been shown to have a lower bone mineral density (BMD) (specifically at the hip and spine, but also for whole body) than age matched controls, as well as an increased fracture risk specifically at the hip and spine (6). The lower BMD (and increased incidence of osteoporosis) may be as a result of the presence of circulating inflammatory cytokines inherent to the disease, the decreased mobility of these patients, resulting in a sedentary lifestyle, or due to certain medications taken to treat the disease such as corticosteroids and methotrexate. Osteoporosis in RA can be generalised, (affecting the axial skeleton such as the hip and lumbar spine), or peri-articular (affecting local areas of inflammation such as the hand joints) in nature. Studies have shown that the majority of bone density loss in RA occurs in the first six months of disease (7). Furthermore, patients with higher RA disease activity have been shown to exhibit a greater loss in BMD, as well as higher indices of bone metabolism compared to those with lower disease activity. Levels of mobility and functional ability have also been correlated with BMD; as have age, stature, and sex independently of RA disease (8), (9).

**People with RA spend large amounts of time in sedentary behaviours.** Bone loss in RA is amplified by the decreased mobility and high levels of sedentary behaviour present in these patients (10). Wolff’s law, states that the skeleton transforms it’s mass and morphology according to individual activity levels and forces placed upon the bone (11). Furthermore, Frost’s mechanostat theory states that each bone has a specific strain threshold, and that in order for that bone to be remodeled, the minimum effective strain must be placed upon it (12). A sedentary individual does not place sufficient strain on the skeleton, and bones are thus remodelled in a direction that promotes bone loss. Patients with RA are therefore at increased risk of osteoporosis due to inflammatory processes inherent to their disease; as well as the consequent effect of a sedentary lifestyle (13). Bone mass in RA can be modified using treatments designed to increase bone mass (such as bisphosphonates, Vitamin D and Calcium), treatments to decrease RA disease activity, or by increasing physical activity sufficiently in order to increase bone loading and remodeling. Patients with RA, however, are known to be more sedentary than their healthy counterparts (10).This sedentary lifestyle is worsened by the joint and muscle damage occurring during severe disease, which results in pain, functional disability and musclular atrophy (14), further decreasing habitual physical activity. Moreover, research has shown that exercises aimed at increasing BMD should be dynamic- comprised of short and vigorous bouts of high impact exercise incorporating rest periods (15). This type of exercise is usually not feasible in patients with a chronic, disabling pain condition such as RA.

Furthermore, RA patients have long been shown to have a decreased health related quality of life (HRQoL) in comparison to the general population (16), which is largely due to the presence of the chronic pain they experience (ref). HRQoL is a relevant measure of disease activity according to the World Health Organisation (17), and studies have shown that “feeling well” is an important patient outcome, along with management of pain, sleep, fatigue, emotional- and physical wellbeing (16). It is thus important to try to limit the amount of pain experienced by patients with RA in order to improve their quality of life and increase their physical activity. Physical activity in patients with RA has been shown to improve sense of wellbeing, decrease morning stiffness, improve sleep patterns, and decrease swollen joint counts over time (18), as well as reducing pain and improving functional ability in these patients (19). These benefits are achieved through mobilization of joints and increasing muscle strength.

Physical activity, as a functional assessment of quality of life can be difficult to assess. Questionnaires and recall diaries are commonly used but are subjective (20), and it is often difficult for patients to recall their activity levels accurately, especially for light to moderate activities (21). Accuracy in these subjective measures also relies on patient fluency in the English language and compliance, and they cannot necessarily be used in all population groups. Accelerometers are growing in popularity as an objective way to measure physical activity, especially in healthy populations (22).

Accelerometers are small, unobtrusive and comfortable, and measure acceleration of the limb to which they are attached by detecting low frequency (0.5-3.2 Hz) gravitational forces (0.05-2.0g) (22). Acceleration is directly proportional to muscle forces generated, which is proportional to energy expenditure (21). This theory along with an inbuilt algorithm allows for the conversion of acceleration into activity counts, which are generated every minute (or in specific time intervals as specified by the user). These counts can be classified into thresholds, indicating light, moderate or heavy intensity levels (21). The Actical device has been shown to be most accurate when placed on the part of the body where the motion occurs, and studies have shown the hip to be the only place able to predict free living activities at all intensity levels, with the wrist and ankle being second and third best respectively (22). Generally, the device needs to be worn for a minimum of ten hours per day in order for that day to be considered valid (23), and at least three valid days of data are required for analysis. Although in many studies 60 continuous minutes of zero activity counts are excluded as non wear time (23); this may not be feasible in an RA population where patients can spend many hours per day being sedentary, therefore continuous zero activity counts may be reflecting extended periods of sedentary time rather than non wear time and thus cannot be excluded.

Acticals have advantages over self-reported measures, such as being able to track intensity, duration and frequency of an activity without relying on patient recall (24). Acticals in particular, can detect varying levels of activity, being able to detect lower level activities and movement in multiple planes (22) and are therefore an ideal tool for measuring physical activity and sedentary behaviour in patients with RA, where most movement is functional and of a low frequency and intensity, and therefore unlikely to be reported accurately using self report measures. Indeed accelerometry has already been used to this effect in other rheumatic diseases (25), as well as in patients with RA (26).

**Traditional exercise interventions may not be feasible in a population with RA.** Exercise interventions making use of light aerobic activity and strength training, as well as stretching, have been conducted in various cohorts of RA patients with varied results, with most studies showing exercise to improve physical fitness and muscle strength with either no change or improved disease activity outcomes (27), however these interventions do not specifically address the problem of low bone mass in these patients.

**Whole body vibration (WBV) is a potential novel exercise intervention for people with RA.** WBV therapy is an exercise whereby a mechanical vibration platform produces energy via forced oscillation. The vibratory waves are then transferred to an individual via propagation through the feet, legs, trunk and finally, the head (28). Studies conducted in postmenopausal women (29), as well as healthy populations (30), and athletes with low BMD (31), have shown WBV therapy to improve BMD, particularly at the hip and spine (32) (33). Although the exact mechanisms whereby WBV therapy increases BMD are unclear, it is likely that there are multiple mechanisms at play. WBV has been shown to activate fluid flow in the caniliculi and lacunae of bone matrix in rats (34), in a manner proportional to loading frequency. This fluid flow creates shear stress on the plasma membrane of osteocytes, bone lining cells, and osteoblasts, which therefore respond accordingly (15). WBV thus activates mechanostransduction in bone and stimulates osteogenesis (34). Furthermore, muscle forces have been shown to exert the greatest osteogenic stimulus on bone, and the generation of these forces through vibration stimulus is likely a contributor to the skeletal adaptations that occur (35). According to Frost’s mechanostat theory, vibration stimulus must sufficiently load bones in order to increase deposition.

WBV has already been used as a means to treat osteoporosis in otherwise healthy populations with low BMD (34), older individuals (36), postmenopausal women (29), athletes at risk of osteoporosis (31), as well as in other diseased populations (37) with mainly positive results including (along with increased BMD), increases in muscle strength, improved proprioception and balance, and decreased pain and fatigue levels. Furthermore, WBV therapy has been shown to increase peripheral blood flow (38) as well as cardiovascular performance (30), and could therefore have an effect on cardiovascular health. Trans et al (2009), used WBV in patients with knee osteoarthritis and found 8 weeks of twice weekly vibration training to significantly improve knee strength in these patients, but did not assess BMD (39). Alentorn-Geli et al (2008) used a dynamic and static WBV protocol, twice weekly for 6 weeks in a group of female patients with fibromyalgia (40). Patients in this study were divided into a control group (who underwent no therapy), an exercise group (who underwent standard RA exercise therapy only), and a WBV group (who underwent WBV training on top of the standard exercise therapy. The authors of this study found that the WBV protocol significantly improved fatigue scores, as well as pain scores in comparison to the exercise group, or the control group, however no measures of BMD were taken. Certain studies have shown WBV therapy to have no effect on BMD in healthy adults (41), or on fatigue or pain levels, balance, or strength (42).

WBV has not, to our knowledge, been used as an exercise intervention for RA, yet it may be a feasible means to increase functional ability in these patients. Since patients with RA are already at risk for developing osteoporosis, and are therefore at greater risk of fracture; WBV could also potentially be a means to attenuate the progressive loss of BMD observed in patients with RA, without the need for a vigorous exercise programme. With this background in mind, the aims of this study are primarily to determine the effects of a WBV programme on functional ability in patients with established RA in comparison to a control group of patients, as well as to determine any effects WBV therapy may have on BMD, disease activity, physical activity levels, HRQoL, or body composition in these patients.

**Methods:**

Study Design:

A semi randomised, single blinded, controlled, two-group parallel design in accordance with the 2010 CONSORT guidelines will be used; with patients being allocated to either group in an alternating manner upon enrollment. Patients in the WBV group will begin a WBV therapy intervention programme for a three month period, while those in the CON group will continue to receive standard care for the three month period. All patients will be assessed at three time points, as follows:


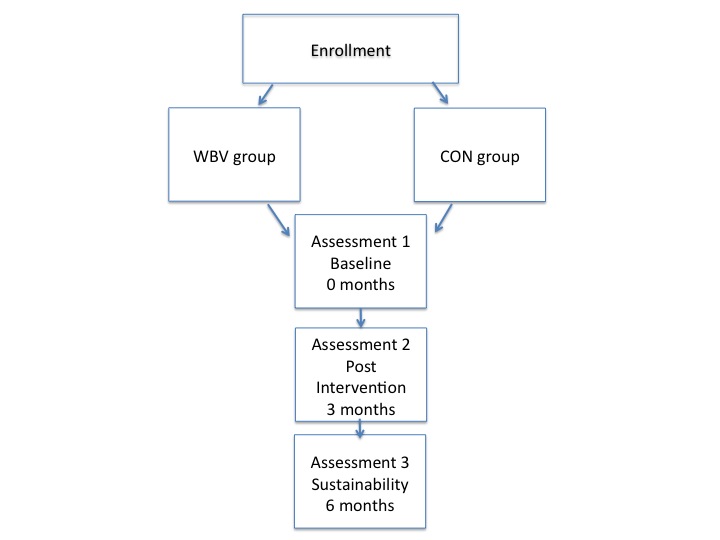


Figure 1. Flow diagram of study design

Participants:

Participants will be recruited from the Rheumatology Clinic at the Chris Hani Baragwanath Academic Hospital in Soweto, South Africa. Recruitment will include the outpatients attending the clinic at the quarterly check-up via a brief interview following dissemination of study information. Consenting patients will be included if they are older than 18 years, have been diagnosed with RA (according to the 1987 ACR criteria (43)) at least three years previously, are on stable drug therapy (prednisone <10mg/day), and had been for at least three months previously. Patients will be excluded if they are HIV+, are using bisphosphonates or corticosteroids, have any co-morbidities that could potentially impact on physical activity levels, are using assistive walking devices, have previously had hip or knee joint replacement surgery, and if they are pregnant.

Ethical Consideration:

This study complies with the international ethical guidelines for a clinical study. Ethical approval has been obtained from the Human Research Ethics Committee of the University of the Witwatersrand (M130113). Patients will be required to read and sign informed consent, and revocation of consent will not detriment the patients in any way.

Trial Registration:

This study has been registered with the Pan African Clinical Trial Registry and has the trial number PACTR201405000823418.

Intervention and control:

All vibration training will be performed on standard power plates (DKN XG 5.0, DKN Technology, California, USA) under the supervision of the primary investigator. Vibration training will consist of 24 total sessions (performed twice weekly for 12 weeks); in intermittent bouts of 60 seconds on the plate and 30 seconds off the plate, repeated 10 times (this protocol was designed to stimulate greater osteogenic responses due to the constant stimulus to the mechanoreceptors (31)). Patients will be required to stand on the plates, barefoot and with knees slightly bent, holding firmly to the bars. Vibration plates will be set at a constant frequency of 30Hz and amplitude of 3mm in order to maximize the osteogenic and muscle activation effects of the therapy (31). The CON group will continue to receive standard care for the intervention period, and will be instructed to continue with their normal daily activities for the three month period.

Sample Size:

A sample size calculation (ß=0.10) showed that at a 5% level (using an SD of 0.19) we would require a sample of 8 participants in each group in order to detect a minimum clinically important difference of 0.22 in HAQ score with a power of 90%.

**Outcome measures:**

All outcomes will be measured at baseline, and reassessed after the three month intervention. The primary outcome of this study will be an improvement in functional ability as assessed by the HAQ. The secondary outcomes will be an attenuation in loss of BMD at the hip, as well as improvements in subjective pain scores, SF-36, CDAI, and objective habitual physical activity levels (Table 1).

Table 1. Summary of outcome measures and respective methodology

| **Outcome** | | **Measurement method** | **Time point (months)** |
| --- | --- | --- | --- |
| **Primary** | Functional Ability | Health Assessment Questionnaire | 0,3,6 |
| **Secondary** | BMD | DXA | 0,3,6 |
|  | Habitual Physical Activity | Accelerometery (Actical worn on the hip) | 0,3,6 |
|  | Disease activity | CDAI assessment | 0,3,6 |
|  | Pain and fatigue | Self reported via Lickert scales | 0,3,6 |
|  | Body composition and anthropometry | DXA, standard scale, and stadiometer | 0,3,6 |

Primary:

Functional Ability:

Patients will be asked to complete the modified Health Assessment Questionnaire (HAQ) (44), which is a RA specific questionnaire that assesses functional ability by providing a score of functionality between 0 and 3, where 0 indicates good functionality and 3 indicates severe functional disability.

Secondary:

The secondary outcomes of the study are an attenuation in loss of BMD, as well as any improvements in HRQoL, disease activity, habitual physical activity or body composition measurements. These will be assessed as follows:

BMD:

All patients will be assessed, for site specific areal BMD at the left hip, lumbar spine (L1-L4), and whole body using Dual X-Ray Absorbtiometry (DXA). T and Z scores will then be calculated according to reference values. All scans will be performed by the same qualified technician on the same machine (Hologic QDR 4500A, Hologic, Boston, USA). The machine is routinely calibrated, and a phantom spine will be scanned daily to determine coefficients of variation of the machine. The technician will be blinded as to the grouping of participants during the study.

Physical Activity:

At each assessment, patients will be fitted with an Actical (Respironics Inc., Murrysville, PA, USA) accelerometer (for the assessment of habitual physical activity) worn on a Velcro belt on the hip for a one week period. Patients will be instructed to wear the accelerometer all day, and to remove the device only while sleeping, bathing or showering. Patients will then return the accelerometer to the clinic one week later. Actical data are recorded in one minute epochs and data are reduced by removing only full days of non-wear time as observed by a full day of zero activity counts. Sleep time is removed by direct observation of the data, and only the remaining data will be considered as wear time. Ten hours of wear time per day and four days of total wear time will be required for inclusion in the analysis (45). Data will be reported as average activity counts per day, as well as percentage of time spent in sedentary, light, moderate and vigorous activity thresholds, as calculated by the inbuilt algorithim on the Actical software. The number of bouts of activity, as well as the number of breaks in sedentary activity per day will also be reported, the methodology of which has been explained previously (46). Participants will be fitted with the same Actical at each visit in order to minimise inter-device variability. This will allow for the assessment of habitual physical activity patterns before and after the intervention.

Disease Activity:

Patients will be assessed for disease activity using the Compound Disease Activity Index (CDAI) (47) which provides a score comprised of tender joint count (TJC), swollen joint count (SJC), patient global assessment (PGA), and physician global assessment (MGA) calculated as follows:

CDAI=TJC+SJC+PGA+MGA

This score allows for the classification of patients according to the severity of their disease where a score <2.8 indicates remission, a score <10 indicates moderate disease activity, a score <22 indicates moderate disease activity, and a score >22 indicates severe disease activity. The physician will be blinded as to the grouping of participants during assessments.

Pain:

At each assessment, patients will be asked to rate their pain levels over the previous week using a Lickert scale ranging from 0-5, where a score of 0 indicates no pain and a score of 5 indicates unbearable pain.

Fatigue:

At each assessment, patients will be asked to rate their fatigue levels over the previous week using a Lickert scale ranging from 0-5, where a score of 0 indicates not feeling tired at all, and a score of 5 indicates the most tired ever felt.

Anthropometry:

Height and weight will be measured to the nearest cm and kg respectively using a standard stadiometer and scale, with patients barefoot and wearing minimal clothing. Thereafter body mass index (BMI) will be calculated.

Body composition:

Body composition, including fat mass and lean muscle mass will be taken from the DXA scan. Percentage body fat and percentage lean muscle mass will then be calculated. Appendicular lean mass (ALM) will also be calculated and used to classify those patients with sarcopenia.

Statistical Analysis:

Statistical analysis will be carried out using Statistica version 12 and Stata version 12/IC 12.0. All data will be presented as mean ± SD, and a p value ≤0.05 is considered significant. Student’s unpaired t-tests will be used to compare HAQ, BMD, physical activity, patient characteristics, and RA disease activity data between the WBV group and CON group at baseline. To assess the effect of the intervention on primary and secondary outcomes, individual linear mixed models will be used for each dependent variable using relevant covariates where necessary. Random intercepts will be used to account for within person correlation of repeated measures. To test a priori hypothesis, the estimated mean scores at each time point (baseline, post-intervention and 6-months) will be contrasted with baseline values. Additionally, the rate of change in dependent variables across each period (baseline and 3 and 3- 6 month maintenance periods) will be compared to each other. Model fit will be assessed using residual plots and diagnostics.

Discussion:

The present study will contribute to the current field of rheumatology by potentially providing a non-pharmacological means to improve functional ability and attenuate the loss of BMD associated with RA. This study may provide a safe, sustainable exercise intervention for these patients that could potentially improve certain aspects of disease activity, as well as HRQoL and habitual physical activity.

The advantages of the present study over previous exercise interventions in RA include, firstly, the use of a novel therapy in RA. WBV therapy has not previously been used in patients with RA (to the best of our knowledge), and could provide a safe and easy form of exercise for patients who are often unable to participate in strenuous activities. Furthermore, the application of an intermittent WBV programme could potentially exhibit the added benefit (over the previous HRQoL and strength benefits seen following WBV therapy in other rheumatic diseases); of attenuating BMD loss in these patients. Very few studies have focussed exercise interventions on improving or attenuating the loss in BMD in this population, despite the very high prevalence of osteoporosis that exists. Usually, interventions designed to increase BMD are dynamic and strenuous, which is not feasible in an RA population. WBV therapy could provide a solution to this problem.

Secondly, the use of an objective measurement of physical activity could further elucidate the benefits of the WBV intervention by providing an accurate, and detailed description of changes that may occur during and following the WBV intervention. Accelerometry allows for the novel examination of changes in patterns of habitual physical activity in this population following the intervention, which will help elucidate which thresholds of physical activity are affected by WBV therapy. Previous research conducted by the authors has shown (using accelerometry for the first time in this population as a means to compare physical activity levels to healthy control participants) that patients with RA are extremely sedentary, and that patients with higher levels of physical activity fare better on certain disease activity and HRQoL outcomes (10). The potential ability of WBV therapy to increase physical activity levels could therefore attribute to any changes seen in functional ability, BMD, disease activity and HRQoL in the present study.

Lastly, the inclusion of a post intervention assessment allows the sustainability of the present protocol to be examined. If any changes are observed in any of the primary or secondary outcomes of the present study, it is important to be able to report on whether these changes will be sustained after cessation of the intervention, thereby adding strength to the feasibility of the intervention.

Funding:

This work was funded by the Connective Tissues Research Grant as well as the National Research Foundation and the Carnegie Large Research Grant.

References:

1. Sokolove J, Strand V. Rheumatoid Arthritis Classification Criteria. Bull NYU Hosp Jt Dis. 2010;68(3):232–8.

2. Ngian G. Rheumatoid arthritis. Austr Family Phys. 2010;39(9):626–8.

3. Kalyoncu U, Dougados M, Daure J. Reporting of patient-reported outcomes in recent trials in rheumatoid arthritis: a systematic literature review. Ann Rheum Dis. 2009;68:183–90.

4. Schneider M, Manabile E, Tikly M. Social aspects of living with rheumatoid arthritis: a qualitative descriptive study in Soweto, South Africa - a low resource context. Health Qual Life Outcomes. 2008 ;6:54.

5. Metsios GS, Stavropoulos-Kalinoglou A, Veldhuijzen van Zanten JJCS, Treharne GJ, Panoulas VF, Douglas KMJ, et al. Rheumatoid arthritis, cardiovascular disease and physical exercise: a systematic review. Rheumatology (Oxford). 2008;47(3):239–48.

6. Haugeberg G, Uhlig T, Falch JA, Halse JI, Kvien TK. Bone mineral density and frequency of osteoporosis in female patients with rheumatoid arthritis: results from 394 patients in the Oslo County Rheumatoid Arthritis register. Arthritis Rheum. 2000;43(3):522–30.

7. Gough AK, Lilley J, Eyre S, Holder RL, Emery P. Generalised bone loss in patients with early rheumatoid arthritis. Lancet. 1994;344(8914):23–7.

8. Laan M. Bone mineral density in patients with recent onset rheumatoid arthritis : influence of disease activity. Ann Rheum Dis. 1993;52:21–6.

9. Deodhar AA, Woolf AD. Bone mass measurement and bone metabolism in rheumatoid arthritis: a review. Br J Rheumatol. 1996;35(4):309–22.

10. Prioreschi A, Hodkinson B, Avidon I, Tikly M, McVeigh JA. The clinical utility of accelerometry in patients with rheumatoid arthritis. Rheumatology (Oxford). 2013;53:1–7.

11. Pearson OM, Lieberman DE. The aging of Wolff’s “law”: ontogeny and responses to mechanical loading in cortical bone. Am J Phys Anthropol. 2004;39:63–99.

12. Turner CH. Three rules for bone adaptation to mechanical stimuli. Bone. 1998 Nov;23(5):399–407.

13. Geraci A, Maria S. Osteoporosis in Rheumatoid Arthritis. 2006;75–93.

14. Ende CHM Van Den, Hazes JMW, Cessie S, Mulder WJ, Belfor G, Breedveld FC, et al. Comparison of high and low intensity training in well controlled rheumatoid arthritis . Results of a randomised clinical trial. Ann Rheum Dis. 1996;55:798–805.

15. Turner CH, Robling a G. Exercises for improving bone strength. Br J Sports Med. 200;39(4):188–9.

16. Wells GA. Patient-Driven Outcomes in Rheumatoid Arthritis. J Rheumatol. 2009;36:33–8.

17. Bazzichi L, Maser J, Piccinni A, Rucci P, Del Debbio A, Vivarelli L, et al. Quality of life in rheumatoid arthritis: impact of disability and lifetime depressive spectrum symptomatology. Clin Exp Rheumatol. 2005;23(6):783–8.

18. Harkcom TM, Lampman RM, Banwell BF, Castor CW. Therapeutic value of graded aerobic exercise training in rheumatoid arthrtitis. Arthritis Rheum. 1985;28(1):32–9.

19. Semanik P, Song J, Chang RW, Manheim L, Ainsworth B, Dunlop D. Assessing Physical Activity in Persons with Rheumatoid Arthritis Using Accelerometry. Med Sci Sport Exerc. 2010;1493–501.

20. Chantler I, Mitchell D, Fuller A. Actigraphy Quantifies Reduced Voluntary Physical Activity in Women With Primary Dysmenorrhea. J Pain. American Pain Society; 2009;10(1):38–46.

21. Pruitt LA, Glynn NW, King AC, Guralnik JM, Aiken EK, Miller G, et al. Use of accelerometry to measure physical activity in older adults at risk for mobility disability. J Aging Phys Act. 2008;16(4):416–34.

22. Kayes NM, Schluter PJ, Mcpherson KM, Leete M, Mawston G, Taylor D. Exploring Actical Accelerometers as an Objective Measure of Physical Activity in People With Multiple Sclerosis. Arch Phys Med Rehabil. the American Congress of Rehabilitation Medicine and the American Academy of Physical Medicine and Rehabilitation; 2009;90(4):594–601.

23. Tudor-Locke C, Camhi SM, Troiano RP. A catalog of rules, variables, and definitions applied to accelerometer data in the National Health and Nutrition Examination Survey, 2003-2006. Prev Chronic Dis. 2012;9:110332. DOI: http://dx.doi.org/10.5888/pcd9.110332

24. Crouter SE, Dellavalle DM, Horton M, Haas JD, Frongillo EA, Bassett DR. Validity of the Actical for estimating free-living physical activity. Eur J Appl Physiol. 2010;DOI 10.1007/s00421-010-1758-2

25. Farr JN, Going SB, Lohman TG, Rankin L, Kasle S, Cornett M, et al. Physical Activity Levels in Patients With Early Knee Osteoarthritis Measured by Accelerometry. Rheumatology. 2008;59(9):1229 –1236.

26. Semanik P, Song J, Chang RW, Manheim L, Ainsworth B, Dunlop D. Assessing physical activity in persons with rheumatoid arthritis using accelerometry. Med Sci Sports Exerc. 2010;42(8):1493–501.

27. Plasqui G. The role of physical activity in rheumatoid arthritis. Physiol Behav. 2008;94:270–5.

28. Wysocki A, Butler M, Shamliyan T, Kane RL. Whole-Body Vibration Therapy for Osteoporosis : State of the Science. Annals Int Med. 2011;155(10):680-686.

29. Bemben DA, Palmer IJ, Bemben MG, Knehans AW. Effects of combined whole-body vibration and resistance training on muscular strength and bone metabolism in postmenopausal women. Bone. 2010;47(3):650–6.

30. Cardinale M, Wakeling J. Whole body vibration exercise: are vibrations good for you? Br J Sports Med. 2005;39(9):585–9.

31. Prioreschi A, Oosthuyse T, Avidon I, Mcveigh J. Whole Body Vibration Increases Hip Bone Mineral Density in Road Cyclists. Int J Sports Med. 2012;33:593–9.

32. Rubin C, Recker R, Cullen D, Ryaby J, McCabe J, McLeod K. Prevention of postmenopausal bone loss by a low-magnitude, high-frequency mechanical stimuli: a clinical trial assessing compliance, efficacy, and safety. J Bone Miner Res. 2004;19(3):343–51.

33. Verschueren SMP, Roelants M, Delecluse C, Swinnen S, Vanderschueren D, Boonen S. Effect of 6-month whole body vibration training on hip density, muscle strength, and postural control in postmenopausal women: a randomized controlled pilot study. J Bone Miner Res. 2004;19(3):352–9.

34. Totosy de Zepetnek JO, Giangregorio LM, Craven BC. Whole-body vibration as potential intervention for people with low bone mineral density and osteoporosis: A review. J Rehabil Res Dev. 2009;46(4):529.

35. Rittweger J. Vibration as an exercise modality: how it may work, and what its potential might be. Eur J Appl Physiol. 2010;108(5):877–904.

36. Kawanabe K, Kawashima A, Sasimoto I, Takeda T, Sato Y, Iwamoto J. Effect of whole-body vibration exercise and muscle strengthening, balance, and walking exercises on walking ability in the elderly. Keio J Med. 2007;56:28–33.

37. Ward K, Alsop C, Caulton J, Rubin C, Adams J, Mughal Z. Low magnitude mechanical loading is osteogenic in children with disabling conditions. J Bone Miner Res. 2004;19(3):360–9.

38. Rauch F. Vibration therapy. Dev Med Child Neurol. 2009;51:166–8.

39. Trans T, Aaboe J, Henriksen M, Christensen R, Bliddal H, Lund H. Effect of whole body vibration exercise on muscle strength and proprioception in females with knee osteoarthritis. Knee. 2009;16(4):256–61.

40. Alentorn-Geli E, Padilla J, Moras G, Lázaro Haro C, Fernández-Solà J. Six weeks of whole-body vibration exercise improves pain and fatigue in women with fibromyalgia. J Altern Complement Med. 2008;14(8):975–81.

41. Torvinen S, Kannus P, Sievänen H, Järvinen T AH, Pasanen M, Kontulainen S, et al. Effect of 8-month vertical whole body vibration on bone, muscle performance, and body balance: a randomized controlled study. J Bone Miner Res. 2003;18(5):876–84.

42. Chanou K, Gerodimos V, Karatrantou K, Jamurtas A. Whole-body vibration and rehabilitation of chronic diseases : A review of the literature. J Sports Sci Med. 2012;11:187–200.

43. Arnett FC, Edworthy SM, Bloch D a, McShane DJ, Fries JF, Cooper NS, et al. The American Rheumatism Association 1987 revised criteria for the classification of rheumatoid arthritis. Arthritis Rheum. 1988;31(3):315–24.

54. Bruce B, Fries JF. The Health Assessment Questionnaire (HAQ). Clin Exp Rheumatol. 2005;23:14–8.

45. Ellingson L, Colbert L, Cook D. Physical Activity Is Related to Pain Sensitivity in Healthy Women. Med Sci Sport Exerc. 2012;DOI:10.1249/MS:1401–6.

46. Tudor-locke C, Brashear MM, Johnson WD, Katzmarzyk PT. Accelerometer profiles of physical activity and inactivity in normal weight, overweight, and obese U.S. men and women. Int J Behav Nutr Phy Act. 2010;7:60. DOI: 10.1186/1479-5868-7-60

47. Aletaha D, Smolen J. The Simplified Disease Activity Index (SDAI) and the Clinical Disease Activity Index (CDAI): a review of their usefulness and validity in rheumatoid arthritis. Clin Exp Rheumatol. 2005;23:100–8.
