# Supplementary material for: In Patients with Established RA, Positive Effects of a Randomised Three Month WBV Therapy Intervention on Functional Ability, Bone Mineral Density and Fatigue Are Sustained for up to Six Months
Source: PLoS One. 2016 Apr 13;11(4):e0153470. doi: 10.1371/journal.pone.0153470 (PMC4830593; doi:10.1371/journal.pone.0153470)
Supplement: S3 Text — (PDF) [file pone.0153470.s004.pdf]

Pan African Clinical Trials Registry

South African Medical Research Council, South African Cochrane Centre
PO Box 19070, Tygerberg, 7505, South Africa
Telephone: +27 21 938 0506 / +27 21 938 0834 Fax: +27 21 938 0836
Email: pactradmin@mrc.ac.za Website: www.pactr.org

**Trial no.:**	PACTR201405000823418	**Date registered:**	2014/05/08
**Trial Status:**	**Retrospective registration - this trial was registered after enrolment of the first participant**		

TRIAL DESCRIPTION

**Public title**	Whole Body Vibration in Rheumatoid Arthritis
**Official scientific title**	Whole Body Vibration in Rheumatoid Arthritis
**Brief summary describing the background and objectives of the trial**	Background: Rheumatoid arthritis (RA) is a chronic autoimmune condition that results in pain and disability. Patients with RA are may be forced into a sedentary lifestyle and, as such, often become predisposed to poor bone health. Patients with RA may also experience a decreased health related quality of life (HRQoL) due to their disease. Whole body vibration (WBV) is a form of exercise that stimulates bone loading through forced oscillation. WBV has also been shown to decrease pain and fatigue in other rheumatic diseases, as well as to increase muscle strength. This paper reports on the development of a semi randomised controlled clinical trial to assess the impact of a WBV intervention aiming to attenuate bone loss, improve functional ability and habitual physical activity levels in patients with RA. Methods and Design: This study is a controlled clinical trial consisting of a cohort of patients with established RA assigned to either a WBV group or a CON (control) group. Patients in the WBV group will undergo three months of twice weekly intermittent WBV sessions, while the CON group will receive standard care and continue with normal daily activities. All patients will be assessed at baseline, following the three month intervention, and six months post intervention. Main outcomes will be an attenuation of loss of bone mineral density (BMD) at the hip and changes in RA disease activity, HRQoL, habitual physical activity levels and body composition. Discussion: This study will provide important information regarding the effects of WBV on BMD in patients with RA, as well as novel data regarding the potential changes in objective habitual physical activity patterns that may occur following the intervention. The sustainability of the intervention will also be assessed.
**Type of trial**	CCT
**Acronym (If the trial has an acronym then please provide)**	
**Disease(s) or condition(s) being studied**	Rheumatoid arthritis, osteoporosis
**Purpose of the trial**	Treatment
**Anticipated trial start date**	2013-08-01
**Actual trial start date**	2013-08-01
**Anticipated date of last follow up**	2014-02-20
**Actual date of last follow up**	2014-02-20
**Anticipated target sample size (number of participants)**	32
**Actual target sample size (number of participants)**	31
**Recruitment status**	Closed to recruitment: follow up complete
**Publication URL**	

Secondary Ids	Issuing authority/Trial register	Links to Secondary ID

STUDY DESIGN

Intervention assignment	Allocation to intervention	If randomised, describe how the allocation sequence was generated	Describe how the allocation sequence/code was concealed from the person allocating the participants to the intervention arms	Masking	If masking / blinding was used
Parallel: different groups receive different interventions at same time during study	Non-randomised		Numbered allocation	Masking/blinding used	Care giver/Provider ,

INTERVENTIONS

Intervention type	Intervention name	Dose	Duration	Intervention description	Group size	Nature of control
Experimental group	Whole body vibration (WBV)	twice per week	12 weeks	Vibration training will consist of 24 total sessions (performed twice weekly for 12 weeks); in intermittent bouts of 60 seconds on the plate and 30 seconds off the plate, repeated 10 times (this protocol was designed to stimulate greater osteogenic responses due to the constant stimulus to the mechanoreceptors). Patients will be required to stand on the plates, barefoot and with knees slightly ben	16	Active
Control group	Control group (CON)	NA	12 weeks	The CON group will continue to receive standard care for the intervention period, and will be instructed to continue with their normal daily activities for the three month period.	15	Active

ELIGIBILITY CRITERIA

List inclusion criteria	List exclusion criteria	Min age	Max age	Gender
Older than 18 years Have been diagnosed with RA (according to the 1987 ACR criteria) at least three years previously On stable drug therapy (prednisone <10mg/day) Had been on stable drug therapy for at least three months previously.	HIV+ Using bisphosphonates or corticosteroids Have any co-morbidities that could potentially impact on physical activity levels Using assistive walking devices Have previously had hip or knee joint replacement surgery Are pregnant.	18 Years	100 Years	Female

ETHICS APPROVAL

Has the study received appropriate ethics committee approval	Date the study will be submitted for approval	Date of approval	Name of the ethics committee
Yes		2013/02/18	University of the Witwatersrand Human Research Ethics Committee

Ethics Committee Address

Street address	City	Postal code	Country
Senate House, 1 Jan Smuts Avenue, Braamfontein	Johannesburg	2000	South Africa

OUTCOMES

Type of outcome	Outcome	Timepoint(s) at which outcome measured
Primary Outcome	Bone mineral density	Baseline Follow up (three months post baseline) Post intervention (six months post baseline)
Secondary Outcome	Functional ability	Baseline Follow up (three months post baseline) Post intervention (six months post baseline)
Secondary Outcome	Physical activity	Baseline Follow up (three months post baseline) Post intervention (six months post baseline)
Secondary Outcome	Health related quality of life	Baseline Follow up (three months post baseline) Post intervention (six months post

		baseline)
Secondary Outcome	Body composition	Baseline Follow up (three months post baseline) Post intervention (six months post baseline)

RECRUITMENT CENTRES				
**Name of recruitment centre**	**Street address**	**City**	**Postal code**	**Country**
Chris Hani Baragwanath AcademicHospital	26 Chris Hani Road, Soweto	Johannesburg	2013	South Africa

FUNDING SOURCES				
**Name of source**	**Street address**	**City**	**Postal code**	**Country**
Connective Tissues Research Grant				
National Research Foundation				
Carnegie Large Research Grant				

SPONSORS						
**Sponsor level**	**Name**	**Street address**	**City**	**Postal code**	**Country**	**Nature of sponsor**
Primary Sponsor	Alessandra Prioreschi	7 York Road Parktown	Johannesburg	2009	South Africa	University

COLLABORATORS				
**Name**	**Street address**	**City**	**Postal code**	**Country**
Mohamed Amin Makda	26 Chris Hani Road, Soweto	Johannesburg	2013	South Africa
Mohammed Tikly	26 chris Hani Road, Soweto	johannesburg	2013	South Africa
Joanne McVeigh	7 York Road Parktown	Johannesburg	2009	South Africa

CONTACT PEOPLE

Role	Name	Email	Phone	Fax
**Principal Investigator**	Ms Alessandra Prioreschi	alessandraprioreschi@ gmail.com	0839890070	

Street address	City	Postal code	Country	Position / Affiliation
7 York Road, Parktown	Johannesburg	2009	South Africa	Researcher

Role	Name	Email	Phone	Fax
**Scientific Enquiries**	Prof Mohammed Tikly	drmohammed@gmail.com	+27119339377	

Street address	City	Postal code	Country	Position / Affiliation
26 Chris Hani road, Soweto	Johannesburg	2013	South Africa	Head of Rheumatology

Role	Name	Email	Phone	Fax
**Public Enquiries**	Ms Alessandra Prioreschi	alessandraprioreschi@ gmail.com	0839890070	

Street address	City	Postal code	Country	Position / Affiliation
7 York Road, Parktown	Johannesburg	2009	South Africa	Researcher

Changes to trial information

Date	Reason	Old Value	Update Value
